# Supplementary material for: Mapping the evolution of fertility support policies in China: A content and instrumental analysis
Source: PLoS One. 2025 Oct 9;20(10):e0332137. doi: 10.1371/journal.pone.0332137 (PMC12510515; doi:10.1371/journal.pone.0332137)
Supplement: S1 Appendix — (ZIP) [file pone.0332137.s001.zip › S1 Appendix. 226 original policy documents/178-中共中央、国务院关于全面加强人口和计划生育工作统筹解决人口问题的决定(FBM-CLI-16-83390).doc]

中共中央、国务院关于全面加强人口和计划生育工作统筹解决人口问题的决定

**发布部门：** [中国共产党中央委员会](https://www.pkulaw.com/law/chl?Aggs.IssueDepartment=a01) [国务院](https://www.pkulaw.com/law/chl?Aggs.IssueDepartment=5)

**发文字号：**中发〔2006〕22号

**发布日期：**2006.12.17

**实施日期：**2006.12.17

**时效性：** [现行有效](https://www.pkulaw.com/law/chl?Aggs.TimelinessDic=01)

**效力级别：** [党内法规制度](https://www.pkulaw.com/law/chl?Aggs.EffectivenessDic=XR12)

**法规类别：** [人口计划管理](https://www.pkulaw.com/law/chl?Aggs.Category=09701) [计划生育管理](https://www.pkulaw.com/law/chl?Aggs.Category=09705)

中共中央、国务院关于全面加强人口和计划生育工作统筹解决人口问题的决定
(2006年12月17日　中发〔2006〕22号)

　　为贯彻党的十六大和十六届三中、四中、五中、六中全会精神，坚持以邓小平理论和“三个代表”重要思想为指导，落实科学发展观和构建社会主义和谐社会的重大战略思想，全面加强新时期人口和计划生育工作，统筹解决我国人口问题，现作出如下决定。
　　**一、**清醒认识全面加强我国人口和计划生育工作的重要性和紧迫性
　　人口问题始终是制约我国全面协调可持续发展的重大问题，是影响经济社会发展的关键因素。我国实行计划生育以来，全国少生4亿多人，提前实现了人口再生产类型的历史性转变，有效地缓解了人口对资源、环境的压力，有力地促进了经济发展和社会进步。实践证明，我国坚持不懈地实行计划生育的基本国策，对建设中国特色社会主义、实现国家富强和民族振兴产生了巨大影响，为促进世界人口与发展发挥了重要作用。
　　当前，我国人口和计划生育工作形势总体是好的。同时，必须清醒地看到，我国人口发展呈现出前所未有的复杂局面，低生育水平面临反弹的现实风险。21世纪上半叶，将迎来总人口、劳动年龄人口和老年人口高峰。今后十几年，人口惯性增长势头依然强劲，总人口每年仍将净增800－1000万人；人口素质总体水平不高，难以适应激烈的综合国力竞争的要求；劳动年龄人口数量庞大，就业形势更加严峻；人口老龄化日益加重，社会保障面临空前压力；出生人口性别比居高不下，给社会稳定带来隐患；流动迁移人口持续增加，对公共资源配置构成巨大挑战；贫困人口结构趋于多元，促进社会均衡发展的任务十分艰巨。总之，人口众多、人均占有量少的国情，人口对经济社会发展压力沉重的局面，人口与资源环境关系紧张的状况，是全面建设小康社会、构建社会主义和谐社会所面临的突出矛盾和问题。
　　实现我国经济社会又好又快发展所面临的重大问题，无不与人口数量、素质、结构、分布密切相关，在人口问题上的任何失误，都将对经济社会发展产生难以逆转的长期影响。以人的全面发展统筹解决人口问题，变人口压力为人力资源优势，为经济社会发展提供持久动力，是实现中华民族伟大复兴的战略选择。全党务必从全面贯彻落实科学发展观的高度，从立党为公、执政为民的高度，从全面建设小康社会和构建社会主义和谐社会的高度，从对中华民族未来发展负责的高度，坚持不懈地做好新时期的人口和计划生育工作。

　　**二、**坚定不移走中国特色统筹解决人口问题的道路
　　我国30多年的人口和计划生育工作实践积累了丰富经验，主要是：必须坚持长期实行计划生育的基本国策，稳定和完善人口政策和生育政策；坚持人口与发展综合决策，充分发挥党和政府在人口和计划生育工作中的主导作用；坚持国家指导与群众自愿、宣传教育与利益导向、行政管理与群众工作、整体推进与分类指导相结合，加强并改进人口和计划生育工作，促进人的全面发展；坚持理论创新、体制创新、管理创新、科技创新，不断提高人口和计划生育工作依法行政、社会管理和公共服务水平；坚持以开放务实的姿态开展国际人口与发展合作交流，树立负责任人口大国的形象。
　　“十一五”时期，人口和计划生育工作进入稳定低生育水平、统筹解决人口问题、促进人的全面发展的新阶段。面对新的形势和任务，既要坚持多年来行之有效的基本经验，又要解放思想、实事求是、与时俱进，研究新情况、解决新问题，不断丰富和发展中国特色统筹解决人口问题的思路、内涵和途径。要全面贯彻落实科学发展观，优先投资于人的全面发展，稳定低生育水平，提高人口素质，改善人口结构，引导人口合理分布，保障人口安全，促进人口大国向人力资本强国转变，促进人口与经济、社会、资源、环境协调和可持续发展。
　　人口和计划生育工作的重点、难点在农村。我国农村生产力还不发达，公共事业发展滞后，社会保障制度不健全，群众生育意愿尚未根本转变，基层基础工作发展不平衡，流动人口管理服务缺乏有效手段，稳定低生育水平面临诸多困难，人口结构性矛盾和深层次问题愈益突出。因此，必须坚持统筹城乡经济社会发展，把农村作为稳定低生育水平、统筹解决人口问题的重中之重，将农村人口和计划生育工作纳入建设社会主义新农村的总体部署，进一步开创我国人口和计划生育工作新局面。

　　**三、**千方百计稳定低生育水平
　　稳定低生育水平是新时期人口和计划生育工作的首要任务，“十一五”期间是实现这一任务的关键时期。综合分析我国经济社会和人口发展趋势，到“十一五”期末，全国人口总量（不含香港、澳门特别行政区和台湾省）要控制在13.6亿人以内；到2020年，人口总量要控制在14.5亿人左右，总和生育率稳定在更替水平以下。
　　为此，必须坚持计划生育基本国策和稳定现行生育政策不动摇，党政第一把手亲自抓、负总责不动摇，稳定人口和计划生育工作机构、队伍不动摇，不断创新人口和计划生育工作体制、机制、手段和方法不动摇。全面加强基层基础工作，坚持依法行政、思想政治教育与利益导向相结合，综合运用法律、行政、教育、经济等手段，建立健全依法管理、村（居）民自治、优质服务、政策推动、综合治理的长效工作机制。
　　建立和完善政府为主、社会补充的人口和计划生育利益导向政策体系。计划生育家庭为国家作出贡献，国家应使计划生育家庭优先分享改革发展成果。全面推行农村计划生育家庭奖励扶助制度和“少生快富”工程，落实独生子女父母奖励、计划生育免费基本技术服务制度。积极探索建立独生子女伤残死亡家庭扶助、长效节育措施奖励、节育手术保险、城市计划生育夫妇年老一次性奖励等制度。对符合社会救助条件的计划生育家庭，通过城乡最低生活保障、医疗救助以及农村五保户供养、特困户生活救助等制度予以帮助。在就业培训、合作医疗、扶贫开发、宅基地划分、改水改厕、沼气应用、新技术推广等方面，各有关部门要制定和完善对计划生育家庭特别是农村独生子女和双女户家庭的优先优惠政策。按照资格确认、资金管理、资金发放、社会监督等职责分设原则，确保每一个符合条件的对象领到奖励扶助金和享受优惠政策。
　　加强思想政治教育。结合“五五”普法，进一步加强人口和计划生育法制宣传，深入开展国策、国情、人口形势教育，积极倡导科学、文明、进步的婚育观念，促进人口和计划生育惠民政策深入人心，引导群众遵纪守法，自觉实行计划生育。
　　坚持依法管理。严肃处理违纪违法行为，凡违法生育的，一律依法征收社会抚养费；造成恶劣影响的，可予以公开揭露；是党员、干部的，依纪依法从严惩处。

　　**四、**大力提高出生人口素质
　　提高出生人口素质，事关千家万户的幸福，事关国家和民族的未来。要科学制定提高出生人口素质的规划及行动计划，加强出生缺陷干预能力建设，全面实施出生缺陷干预工程，实行定期评估、通报制度。人口和计划生育技术服务机构与医疗保健机构要在各自职责范围内密切配合，大力宣传和普及预防出生缺陷科学知识，实施计划生育生殖健康促进计划，加强婚育咨询和指导，积极开展婚前和孕前保健、孕产期保健、产前筛查和诊断、产后访视、新生儿疾病筛查和康复等工作；促进住院分娩和母乳喂养，为贫困妇女提供必要的生育救助和安全接生。
　　倡导科学婚检。加强性病和艾滋病防治工作，预防艾滋病母婴传播。对影响出生缺陷的生物遗传、社会环境、不良生活方式等重大危险因素进行研究、评估和干预。大力普及婴幼儿抚养和家庭教育的科学知识，开展婴幼儿早期教育。强化独生子女社会行为教育和培养。

　　**五、**综合治理出生人口性别比偏高问题
　　出生人口性别比过高、持续时间过长，必然影响社会稳定，关系到广大人民群众的切身利益。要建立党政负责、部门配合、群众参与的标本兼治工作机制，加强综合治理的过程评估和责任考核。深入开展“关爱女孩行动”、“婚育新风进万家”活动。以消除性别歧视为重点，广泛宣传男女平等、少生优生等文明婚育观念，普及保护妇女儿童权益的法律法规知识。制定有利于女孩健康成长和妇女发展的社会经济政策，促进男女平等就业和共同参与社会经济活动。对农村计划生育女儿户给予奖励，在扶贫济困、慈善救助、贴息贷款、就业安排、项目扶持中对计划生育女儿户予以倾斜，推动“幸福工程”、“春蕾计划”等社会公益活动。鼓励男到女家落户，依法保护妇女的宅基地、房屋等继承权和土地承包权等权益。
　　严禁非医学需要的胎儿性别鉴定和选择性别的人工终止妊娠。建立B超检查和人工终止妊娠登记、孕情检测、孕产过程管理等制度。完善执业资质认证和B超使用准入制度。对终止妊娠药品和促排卵药品实行严格的处方管理。运用法律手段，严厉打击非法实施胎儿性别鉴定和选择性别人工终止妊娠的行为，依法严惩溺、弃、残害女婴和拐卖、绑架妇女儿童的犯罪活动及歧视、虐待生育女婴的妇女等违法行为，保障妇女儿童合法权益。实施举报制度，加强社会监督。

　　**六、**不断完善流动人口管理服务体系
　　我国人口流动规模庞大。做好流动人口管理和服务，实现人口有序流动和合理分布，关系改革发展稳定大局，关系低生育水平长期稳定。要深化流动人口管理服务体制改革。逐步改变城乡二元结构，建立城乡统一的人口登记制度，健全出生人口登记和生命统计制度，实行流动人口居住证制度。将流动人口管理服务纳入地方经济社会发展规划，促进流动人口融入城市生活。解决流动人口在就业、就医、定居、子女入托入学等方面的实际困难，逐步将进城务工人员纳入社会保障体系，保护其合法权益。
　　建立流动人口计划生育统一管理、优质服务新体制。将流动人口纳入流入地人口总数，实行以流入地为主的目标管理双向考核。完善流动人口计划生育管理机构和服务网络，配备必要的社区计划生育专（兼）职人员。流入地按照“属地化管理、市民化服务”的原则，将流动人口计划生育管理服务纳入经常性工作范围，提供与户籍人口同等的免费服务。把进城务工人员计划生育管理服务经费纳入各级财政预算。强化社区流动人口登记制度，实现流动人口信息适时变动、异地查询和跟踪管理。相关部门为流动人口办理经商、务工、购房、租房、社会保障等手续时，应与人口和计划生育部门密切配合、互通信息。在流动人口集中的社区、企业、集贸市场等成立计划生育协会。流出地要配合流入地，做好外出人员的宣传培训、免费办理婚育证明等相关工作。

　　**七、**积极应对人口老龄化
　　目前，我国已进入老龄社会，60岁及以上老年人口达1.44亿人，占总人口的11.03％。要制定和落实老龄事业发展战略规划和政策，把逐步建立覆盖城乡居民的养老保障制度作为社会保障体系建设的重点，构建以居家养老为基础、社区服务为依托、机构照料为补充的养老服务体系。
　　农村要探索建立多种形式的计划生育家庭养老保险制度。有条件的地方，可建立政府、集体和社会共同参与的养老服务机构。对生活不能自理的农村计划生育家庭老年父母，按规定提供适当补助。对军烈属、鳏寡及其他有特殊困难的老年人，按规定给予养老救助。
　　城市要逐步完善社会统筹与个人账户相结合的基本养老保险制度，构建多层次的城镇养老保障体系。积极发展适合老年人特点的知识和经验密集型服务业，为老年人提供力所能及参与社会的机会。提高养老服务机构在城市规划中的比重，发展社区老年活动场所和服务设施，制定优惠政策，鼓励社会开办各种类型的养老服务机构。
　　发扬敬老、养老、助老的良好社会风尚，积极探索和实施“爱心护理”等工程。从老年预防保健入手，倡导健康生活方式，营造出行安全和起居方便的环境。探索建立老年服务志愿者、照料储蓄、长期护理保险等社会化服务制度。大力弘扬子女赡养、家庭养老和邻里互助的传统美德。要加强舆论监督，对拒绝赡养或虐待父母的行为，追究法律责任。大力发展老龄产业，建立满足特殊需求的老年用品和服务市场。

　　**八、**切实加大人口和计划生育事业保障力度
　　人口和计划生育事业是政府履行社会管理和公共服务职能的重要组成部分。对人口和计划生育事业的公共投入，是保稳定、促发展的基础性投入。要从财政、基础设施、人力、科技等方面加大投入，确保人口和计划生育事业持续健康发展。
　　建立稳定增长的投入保障机制。人口和计划生育财政投入增长幅度要高于经常性财政收入增长幅度，确保法律法规规定的各项奖励优惠政策、县乡人口和计划生育技术服务机构基本建设和队伍建设、计划生育经常性工作、计划生育免费基本技术服务等经费的落实。各级财政要逐年增加投入，到2010年，全国人均人口和计划生育事业费在“十五”期末人均10元的基础上增加到22元，继续安排“十五”期间已将社会抚养费、乡（镇）统筹费纳入财政预算的人口和计划生育事业费支出人均8元，届时，各级财政投入人口和计划生育事业费达到人均30元。鼓励企业、社会团体和个人向人口和计划生育事业投入。积极运用市场机制，采取建立基金、开发险种等方式，吸引国内外资金。加强监督检查，严肃查处在奖励优惠资金发放和社会抚养费征收、管理、使用中的违纪违法行为。
　　强化人口和计划生育管理服务体系。形成特色鲜明的行政部门、服务机构、自治组织、群众团体目标一致、上下互动、信息共享、运转高效的科学管理格局。切实加强面向基层的服务体系建设，国家重点支持中西部县（市、区）、乡（镇）中心服务站和流动服务车建设。利用网络优势，面向健康、亚健康人群，履行宣传教育、技术服务、信息咨询、药具发放和人员培训等职能。实施“新农村新家庭计划”和“生育关怀行动”，开展计划生育、生殖健康及家庭保健等服务。
　　加强人口和计划生育队伍职业化建设。完善人口和计划生育专业技术人员职称制度，探索建立人口和计划生育专业技术人员、专业工作人员职业资格证书制度，力争“十一五”期间形成结构合理的专业技术队伍。实行绩效考核，完善人员准入、选拔任用、引进培养、交流、退出机制。
　　加快推进人口和计划生育信息化建设。充分发挥从中央到省、市、县、乡、村人口和计划生育信息网络作用，开发和运行人口宏观调控管理、人口发展趋势预测、人口安全预警预报、基层管理服务、流动人口综合管理、奖励优惠信息管理等综合应用系统，完善人口数量、素质、结构、分布、迁移、就业、贫困等关系国民经济和社会发展重大问题的信息资源建设和开发利用，提高人口和计划生育科学决策、社会管理和公共服务水平。
　　积极推动计划生育生殖健康科技创新。建立以公益类研究机构为主体的公共科技服务体系，依托高等学校和重点科研机构，建设具有国际先进水平的计划生育生殖健康科研基地和学科体系，组建若干多学科交叉的国家重点实验室和工程技术研究中心。充分发挥我国传统中医药优势，运用生物技术、信息技术和新材料技术等高科技成果，开展计划生育生殖健康领域重点课题的联合攻关，力争取得突破性进展。大力发展计划生育生殖健康产业。

　　**九、**进一步加强对人口和计划生育工作的领导
　　新时期人口和计划生育工作任重道远。各级党政领导干部必须充分认识稳定低生育水平的长期性和艰巨性，充分认识统筹解决人口问题的复杂性和紧迫性，充分认识经济社会发展为人口和计划生育工作带来的战略机遇和有利条件，坚决克服盲目乐观和麻痹松劲情绪，切实增强做好人口和计划生育工作的坚定性、主动性和创造性。
　　建立健全统筹解决人口问题的决策与调控机制。各级党委和政府要把人口和计划生育工作摆上重要议事日程，研究解决重大问题，部署重要任务，加强统筹协调和督促检查。将人口发展战略和规划纳入国民经济和社会发展总体规划。制定人口发展评估体系，完善人口发展和人口安全预报预警制度，监控人口发展规划执行情况。进一步完善地方各级人口和计划生育工作领导机构和协调机制。完善目标管理责任制，对党政领导、责任部门、人口和计划生育部门分别进行考核，实行“一票否决”制度。坚持把人口和计划生育工作纳入中央重大事项督查范围。各省、自治区、直辖市党委和政府每年要将本地区人口和计划生育工作情况向中央作专题报告。改革管理体制，转变政府职能，进一步落实机关、企事业、社会团体法定代表人的人口和计划生育工作责任制，充分发挥计划生育协会等群众团体在基层人口和计划生育工作中的生力军作用。
　　全面加强人口和计划生育法制建设。进一步完善法律法规体系，严格贯彻落实《[中华人民共和国人口与计划生育法](https://www.pkulaw.com/chl/252f7ce51132230bbdfb.html)》等法律法规，认真总结行之有效的成功经验和管理制度，适时修订或制定有关法律法规。切实提高依法行政水平，坚持执法为民，实行政务公开，尊重群众的知情权、参与权、监督权，保障群众计划生育合法权益。落实人口和计划生育行政执法责任制，对违纪违法行政造成严重后果的，给予党纪、政纪处分，直至追究法律责任。
　　稳定健全基层工作机构和队伍。在农村综合改革和城市行政区划调整中，确保人口和计划生育工作机构和人员稳定，不得随意撤并和改变机构性质。加快人口和计划生育管理体制改革。按照常住人口规模比例配备人口和计划生育工作人员。县乡两级人口和计划生育工作岗位特殊、任务艰巨，要按规定落实基层工作人员的工资和社会保障待遇。人口和计划生育技术服务机构从事公益服务，经费由财政保障。选拔德才兼备的优秀人才，充实、配强人口和计划生育部门领导班子。对政绩突出的优秀干部要注意培养和使用。
　　加强人口和计划生育宣传教育。报刊、广播、电视、互联网等大众传媒，特别是主要媒体要制定规划，采取灵活多样、生动活泼的形式，持续广泛开展人口和计划生育方针政策的宣传，总结报道先进经验和典型，扩大宣传的覆盖面和影响力。中等以上学校要将人口和计划生育、生殖健康纳入相关课程教学内容或开设专题讲座等。利用城市社区、农村基层各种文化场所和宣传途径，开展群众喜闻乐见的人口文化活动。
　　加强人口与发展领域的国际交流与合作，扩大南北对话，促进南南合作，建立相互尊重、共同发展的伙伴关系，争取国际社会广泛理解和支持。积极参与人口与发展领域国际援助和国际规则的制定。遵循国际人口与发展大会行动纲领和联合国千年宣言精神，进一步促进人权事业发展，提高人权保障水平。
　　中国人民解放军和中国人民武装警察部队的人口和计划生育工作，参照本决定精神，由解放军和武警部队另行制定。

© 北大法宝：（[www.pkulaw.com](https://www.pkulaw.com/)) 专业提供法律信息、法学知识和法律软件领域各类解决方案。北大法宝为您提供丰富的参考资料，正式引用法规条文时请与标准文本核对。
欢迎查看所有[产品和服务](https://www.pkulaw.com/helps/2.html)。法宝快讯：[如何快速找到您需要的检索结果？](https://www.pkulaw.com/helps/70.html)[法宝](https://www.pkulaw.com/helps/69.html)V6有何新特色？

扫描二维码阅读原文

**原文链接：**<https://www.pkulaw.com/chl/5e7b01010a4102ffbdfb.html>
